# Little Ones in the Dental Chair

**Published:** 1901-12-15

**Authors:** Clark R. Rowley


### LITTLE ONES IN THE DENTAL CHAIR.

BY CLARK R. ROWLEY, D.D.S.

Read at the Bi-State Convention of the Northern Indiana and Southwestern Michigan Dental Societ'es at Goshen, Ind., S-ptember 25, 1901.

A paper written and read by Prof. G. E. Johnson, of Andover, Mass., before the American Academy of Dental Science, February 6, 1901, at Boston, and published in the July number of the *International Dental Journal*, prompted me to write a paper for your consideration on the above named subject. I trust it may meet with your approval and call forth such discussion as the subject deserves.

Prof. Jonson believes, and cites us to articles written by Prof. James Leon Williams, of England, for further evidence, that with the advancement of civilization there has come an increasing tendency toward physical degeneracy, more especially noticeable in regard to the jaws and teeth of the present generation. Now, if Prof. Johnson's argument is correct, and he gives us figures and data in proof of his theory, I say the time has arrived and long passed when the dental profession should make it a paramount issue, and by strenuous effort try to combat the abnormal condition pictured to us by Prof. Johnson.

I believe the tendency has been, and is to-day, on the part of the dental profession, to avoid as much as possible operations for children. While we have the specialist in crown and bridge work, prosthetic and operative work, extracting, etc., never in all my thirty years of experience have I found a dentist devoting his time and study in caring for children's teeth. Is it because the child's teeth do not require our attention? or is it because we fail to get the same results operating for the child that we do in operating for the adult? I believe the trouble is to a great extent in ourselves, by following a line of practice never intended for children. The rubber dam, gold filling with contact points, and modern cavity preparation should never be used for the trembling child. Only where entire confidence and control of the child can be had is it possible for us to accomplish anything along the line of parmenence; and in order to obtain this requisite on the part of the little ones in the dental chair it is necessary to begin very early in life. I try to impress upon the mother the importance of allowing the dentist to examine the mouth of the baby as early as the tenth week of life, that any deformities may be found if they exist, and proper instructions given as to remedial agents. Also the importance of continuing the mouth bath up to the age of two and a half years, when the brush should take the place of the previously used sponge or linen cloth. Insist that the brush be used two or three times each day. By this method, if adopted and continued up to the sixth year, when the first permanent inferior incisor should make its appearance, we shall then have a little one that will automatically come, through choice, to have done whatever may be necessary for the proper preservation of the teeth.

All babies are not lovers of dentists, but all dentists should be lovers of babies. If you are the family dentist you ought to be a welcome visitor to the household, that you may the more readily form an acquaintance with the little ones, and by instinct they will soon learn if you care for them. After the acquaintance has ripened they more readily accede to your suggestions when you have them in the dental chair.

Prof. Johnson contends that a very great majority of even well-to-do parents do not employ a dentist to care for the babe's teeth, and says that therefore few dentists really come in touch with a representative body of children. I believe this to be largely the fault of the dental profession. As all have felt an incompetency in caring for the little one as we should for him at the age of maturity, so as a profession we have tried to avoid child practice; hence this deplorable condition of children's teeth as reported by Prof. Johnson. The importance of the subject is more forcibly impressed upon our minds by reading some of Prof. Johnson's published statistics. I trust you will bear with me while I quote a few of them.

In 1880 Dr. Samuel Sexton, aural surgeon to the New York Eye and Ear Infirmary, made a thorough examination of the teeth of eighty school children. Scarcely any of them were free from dental irritation.

Mr. Denison Pedley in England conducted an examination of the teeth of 3,800 school children from three to sixteen years of age. Seventy-five percent had diseased teeth ; and, while this was considered bad, it is much better than the condition of American children thus far examined.

Unghavari, in Hungary, examined 1,000 children between the ages of six and twelve, and found that over 87 percent had diseased teeth.

In Hamburgh upon examination it was found that 94 percent of 335 children had diseased teeth.

A very extensive examination of the teeth of school children has been made in Prussia. In nineteen different cities 19,725 children were examined. Ninety-five percent of the children from six to fifteen years of age were afflicted with caries, and only 218 children of the nineteen thousand and over had ever been treated by a dentist.

In this country, in Andover, Mass., 497 school children were examined, 257 boys and 240 girls, from four to eighteen years of age. Nearly 97 percent of all the children were afflicted with caries.

Prof. Johnson's paper contains many more statistics too numerous to mention, but all along the line of juvenile degeneracy and all tending to place within our view as dentists the deplorable condition of children's teeth as they exist to-day and the very great importance of formulating some mode of dental child practice that will change this abnormal condition of children's teeth to a more normal and healthy state. With this cry coming from all parts of the globe for dentistry pure, simple and scientific, is it any wonder that thousands are flocking into the profession, and that colleges professing to teach the dental art are conceived and prematurely given birth almost every month in the year? And yet with the growth of the profession numerically the teeth of the rising generation grow worse, and as a profession we seem to be holding out the glad hand to welcome this unsavory condition of children's teeth that we may be enriched by the coming harvest and our skill exploited by a display of the bridge, crown and porcelain inlay. But let us keep within the limit of our subject.

Prof. Johnson says there is great need of a motive on the part of the people to care for their own teeth and the teeth of their children, and that there is a deplorable ignorance and inappreciation of the value of good teeth and the harm arising from their neglect.

The discussion of this paragraph might more consistently come under the head of public education in the care of teeth. But I would say the motive is great enough when health and beauty are destroyed by diseased teeth, and I can not believe it is so much ignorance and inappreciation as it is a dread of the operation and a want of proper instruction to the parents in advising them in the care of the little ones' teeth. And if you will bear with me I would like to add to what I have already said a few words further in regard to the care of children's teeth. As previously stated, do not expect to operate for the child, when operations are necessary, as you would for the man or woman. First let us strive to avoid decay in children's teeth. Second, when found, apply the remedy with as little discomfort as possible. Absolute cleanliness, and mild, pleasant antiseptics are almost specifics against decay. Formaldehyde and silver nitrate, when properly applied, will arrest decay and save the filling of deciduous teeth.

Foods and drinks should enter largely into our advice to women about to become mothers. When the stomach is refractory, as is many times the case in pregnancy, prescribe Horlick's malted milk and lime water. At the age of eighteen months the child should be fed liberally of cow's milk diluted with barley water. At the age of two and a half and three the child should be fed liberally of rolled oats, rolled wheat, or graham bread, meat, fish, eggs and fruits. Soft or rain water should not be used for drinking purposes, Hard water rich in lime should be advised. The chewing of bread crusts, grape nuts, and masticating broiled round-steak is good exercise for the teeth on and after the age of seven and eight years. I would suggest osteopathy and massage for contracted arches, also for prognathous upper or lower jaw. Much can be accomplished in either case by gently yet thoroughly manipulating the parts several times a day (this the mother or nurse can be properly instructed in,) and thereby save expensive and tedious regulating cases later in life.

Now, gentlemen, I leave the subject in your hands. A word, however, I would impress upon you as to the importance of this great study. Let the fathers and mothers see that you are enough interested in the welfare of their children's teeth to freely offer advice, not always with the expectation of being remunerated financially. And at all times let us remember to think of the child professionally very early in life ; and I believe if we do not live to receive their thanks when they have reached the age of maturity, we will have their expressions of good will long after we have passed into the eternal shadow.

